# PM2RA: A Framework for Detecting and Quantifying Relationship Alterations in Microbial Community

**DOI:** 10.1016/j.gpb.2020.07.005

**Published:** 2021-02-11

**Authors:** Zhi Liu, Kai Mi, Zhenjiang Zech Xu, Qiankun Zhang, Xingyin Liu

**Affiliations:** 1 State Key Laboratory of Reproductive Medicine, Center of Global Health, Nanjing Medical University, Nanjing 211166, China; 2 Department of Pathogen Biology-Microbiology Division, Key Laboratory of Pathogen Biology of Jiangsu Province, Nanjing Medical University, Nanjing 211166, China; 3 Key Laboratory of Human Functional Genomics of Jiangsu Province, Nanjing Medical University, Nanjing 211166, China; 4 School of Food and Technology State, Key Laboratory of Food Science and Technology, Nanchang University, Nanchang 330031, China; 5 Key Laboratory of Holistic Integrative Enterology, Second Affiliated Hospital of Nanjing Medical University, Nanjing 210003, China

**Keywords:** Microbiome, Microbial relationship alteration, Profile monitoring, Network, Human disease, Microbial community

## Abstract

The dysbiosis of gut microbiota is associated with the pathogenesis of **human diseases**. However, observing shifts in the microbe abundance cannot fully reveal underlying perturbations. Examining the relationship alterations (RAs) in the **microbiome** between health and disease statuses provides additional hints about the pathogenesis of human diseases, but no methods were designed to detect and quantify the RAs between different conditions directly. Here, we present **profile monitoring for microbial relationship alteration** (PM2RA), an analysis framework to identify and quantify the microbial RAs. The performance of PM2RA was evaluated with synthetic data, and it showed higher specificity and sensitivity than the co-occurrence-based methods. Analyses of real microbial datasets showed that PM2RA was robust for quantifying microbial RAs across different datasets in several diseases. By applying PM2RA, we identified several novel or previously reported microbes implicated in multiple diseases. PM2RA is now implemented as a web-based application available at http://www.pm2ra-xingyinliulab.cn/.

## Introduction

The gut microbiome is considered as the second genome of human body and is linked to many human diseases [1], [2], [3]. The central goal of human microbiome studies is to identify microbes associated with diseases. The identified bacteria can provide insights into disease etiology and be potential biomarkers for disease diagnosis and prevention. Furthermore, they could be therapeutic targets if verified as causal factors for certain diseases.

The development of next-generation sequencing technologies enables culture-independent investigations of the human microbiome’s role in health and disease via direct DNA sequencing. Both 16S rRNA sequencing and metagenomic sequencing have been used to study the human microbiome, allowing the creation of a table for the differential abundance analysis of microbes under various biological conditions [4], [5], [6]. Differential abundances of certain microbes may contribute toward conferring a specific trait in a given situation. However, focusing on individual microbe alterations while ignoring potential relationship alterations (RAs) limits reflection on the real perturbation of ecological networks under phenotype changes.

The human microbiome is a complex bacterial community in which sub-communities are formed based on shared niche specializations and specific interactions between individual microbes. The mutual associations within the residing microbial communities play an important role in the maintenance of eubiosis [7], [8], [9]. Bacteria can interact with each other in numerous ways, such as commensalism, mutualism, and competition, which can have neutral, beneficial, and detrimental effects on the microbes involved, respectively. Commensalism refers to situations where some constituent microbes of an ecosystem derive benefits from other members without helping or harming them. Mutualism describes interactions that benefit all organisms involved [8]. A bacterium might also directly compete with another one for the same nutrition source, thereby creating a competition [7], [10]. These kinds of functional relationships are referred to as profiles, which can be linear, polynomial, nonlinear, or a waveform [11]. The disruption of these relationships can lead to disorders in the microbial community structure, furthering dysbiosis.

Many studies have modeled microbiome profiles with a linear correlation between two types of microbes [12], [13], [14], and co-occurrence networks are constructed to describe the whole microbial communities. Based on these co-occurrence networks, alignment-based [15], [16], [17], [18] or alignment-free [19], [20], [21] methods have been proposed to visualize the RAs between different conditions, such as health *vs.* disease. The alignment-free network comparison methods aimed to quantify the overall topological difference between networks, irrespective of node mappings between the networks and without identifying any conserved edges or subgraphs. The alignment-based methods aimed to find a mapping between the nodes of two networks that preserves many edges and a large subgraph between the networks. These strategies can neither quantify the association changes in a specific group of microbes nor pinpoint the exact nodes that contribute to the community differences between two conditions.

Applying the concept of profile monitoring, which is widely used to monitor the relationship consistency between variables in the food-production, manufacturing, and healthcare industries [22], we developed an innovative analysis framework called profile monitoring for microbial relationship alteration (PM2RA) to detect and quantify the profile alterations within microbial communities under various conditions. To our knowledge, PM2RA is the first method to make direct comparisons of microbial associations between conditions without initially constructing co-occurrence networks. By testing both synthetic and real datasets, we demonstrate that PM2RA is high in sensitivity and specificity, and identifies both previously identified and novel microbes involved in multiple diseases. Moreover, PM2RA is robust in identifying important microbes in datasets obtained from different cohorts and different sequencing strategies. A web-based implementation of PM2RA is available at http://www.pm2ra-xingyinliulab.cn/.

## Method

### PM2RA framework

**Figure 1:**
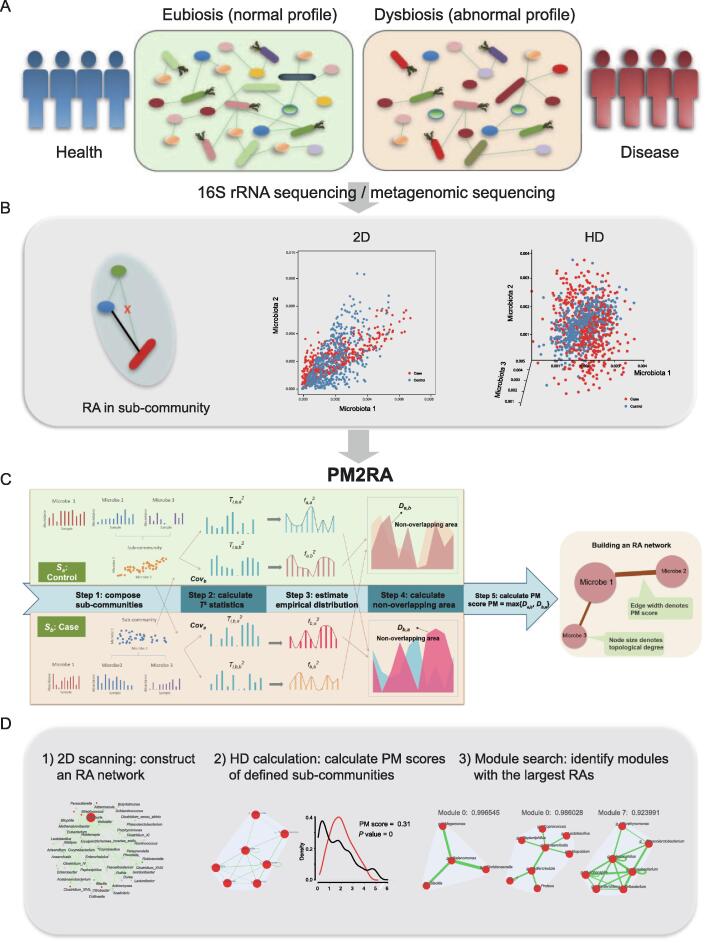
**Overview of the PM2RA method** **A.** Dysbiosis of gut microbiota involves disturbed microbe relationships under human disease conditions. **B.** A relationship change can involve two or more microbes in a 2D or HD level. **C.** The PM2RA methodology framework. First, compose the sub-communities each consisting of two microbes. Second, calculate the *T*^2^ statistic for each sub-community. Third, estimate the empirical distribution of the *T*^2^ statistics. Fourth, calculate the non-overlapping area between distributions. Finally, calculate the PM score for each sub-community. **D.** The PM2RA has developed with three methods: 1) 2D scanning for pairwise RAs among the microbial community between two conditions, 2) HD calculation by which the PM score of any defined sub-community with two or more microbes could be calculated, and 3) module search based on the HD calculation. PM2RA, profile monitoring for microbial relationship alteration; 2D, two-dimensional; HD, high-dimensional; PM, profile monitoring; RA, relationship alteration.

The human microbiome is a complex bacterial community where the relationships between microbes play important roles in the maintenance of eubiosis (Figure 1A). Examining the RAs in the microbiome between health and disease conditions provides additional insights into the pathogenesis of human diseases (Figure 1B). PM2RA is specifically designed to quantify the RA(s) involving two or more microbes under different conditions. The basic idea of PM2RA analysis is to project the abundance data of two or more microbes under two conditions into the same space via Hotelling’s *T*^2^ statistic, and compare the difference in the distribution of *T*^2^ statistics to represent the RAs between two conditions. We developed a new scoring scheme called PM (profile monitoring) score to quantify the RA of each sub-community under different conditions in five steps (Figure 1C). The more the sub-community alters, the bigger the PM score is. Next, we built an RA network in which edges denote the corresponding PM scores (Figure 1C). The framework comprises the following steps.

#### *Compose the sub-communities*

In a microbiota profile, every two microbes and the interaction between them are defined as a sub-community. PM2RA quantifies all possible sub-community RAs and outputs the RA network.

#### *Calculate the T^2^ statistics*

Hotelling’s *T*^2^ statistic is one of the most popular statistics for monitoring the variables of a multivariate process [23]. This statistic considers both the mean value and covariance matrix, which makes it suitable for reducing two-dimensional (2D) or high-dimensional (HD) microbial data into one-dimensional data containing both abundance and relationship information. The *T^2^* statistic is the multivariate counterpart of the *t* statistic and is widely used in multivariate processes for consistency monitoring in both industry and biology [24], [25]. It can be viewed as the generalized distance between the observed vector $\overset{-}{x}$ and the mean vector *μ* weighted by the inversion of the covariance matrix, ${\left(\overset{-}{x}-\mu \right)}^{\text{'}}\Sigma^{-1}\left(\overset{-}{x}-\mu \right)$ [26]. Since both μ and Σ are involved in the calculation, *T*^2^ statistic is sensitive to both relative abundance change and relationship change.

Let $S=\left\{S_{a},S_{b}\right\}$ denote the condition set in which microbe RAs are interested, such as $S=\left\{S_{a}=health,S_{b}=disease\right\}$. To guarantee symmetry, that is, the RAs observed from the condition $S_{a}$ to $S_{b}$ are equal to those from the condition $S_{b}$ to $S_{a}$, four types of *T^2^* statistics for each sub-community are calculated as follows:

$${T_{i,a,a}}^{2}={\left(x_{i,a}-X_{a}\right)}^{\text{'}}{{COV}_{a}}^{-1}\left(x_{i,a}-X_{a}\right),\;\;\;\;i=1,2,\cdots N_{a}$$

$${T_{i,b,a}}^{2}={\left(x_{i,b}-X_{a}\right)}^{\text{'}}{{COV}_{a}}^{-1}\left(x_{i,b}-X_{a}\right),\;i=1,2,\cdots N_{b}$$

$${T_{i,a,b}}^{2}={\left(x_{i,a}-X_{b}\right)}^{\text{'}}{{COV}_{b}}^{-1}\left(x_{i,a}-X_{b}\right),\;i=1,2,\cdots N_{a}$$

$${T_{i,b,b}}^{2}={\left(x_{i,b}-X_{b}\right)}^{\text{'}}{{COV}_{b}}^{-1}\left(x_{i,b}-X_{b}\right),\;i=1,2,\cdots N_{b}$$

$X_{a}$,$X_{b}$ and ${COV}_{a}$,${COV}_{b}$ are the mean relative abundance vectors and the covariance matrices of microbes under conditions $S_{a}$ and $S_{b}$, respectively. $x_{i,a}\;\;\mathrm{and}\;\;x_{i,b}$ denote the relative microbial abundance of the i^th^ sample under $S_{a}$ and $S_{b}$, respectively.

In the pairwise RA analysis, the number of microbes is two, and the sample size is usually much larger than that. In this case, the possibility of strict collinearity between two microbes is low, so we can assume that the covariance matrix is nonsingular.

#### *Estimate the empirical distribution of the T^2^statistics*

The probability density function is an informative, descriptive tool and can reflect the mean, standard deviation, and other statistical properties of the dataset. A straightforward way to calculate alterations between two datasets is to compare their probability density functions. The *T*^2^ statistic follows a scaled chi-squared distribution under the assumption that samples have a normal distribution, although this assumption is usually violated in the microbiota abundance context. Researchers believe that the normal distribution is not a good descriptor of the microbiota sequencing data. Instead, zero-inflated negative binomial models are usually recommended for handling excessive zeros in such data [27]. The *T^2^* statistic of the microbe community certainly does not follow the chi-squared distribution. Thus, PM2RA uses a kernel distribution to represent the probability density of the *T*^2^ statistics derived for each sub-community. The estimated kernel distribution produces a non-parametric, smooth, continuous probability curve that adapts itself to the data, rather than selecting a density with a particular parametric form (*e.g.*, a chi-squared distribution) and estimating the parameters. More straightforwardly, the kernel density estimation method imposes no parametric assumptions on the underlying distribution function. In this step, the kernel estimation method proposed by Scott [28] is applied to ${T_{i,a,a}}^{2}$, ${T_{i,b,a}}^{2}$, ${T_{i,a,b}}^{2}$, and ${T_{i,b,b}}^{2}$. The estimated empirical probability density functions are denoted as ${f_{a,a}}^{2}$, ${f_{b,a}}^{2}$, ${f_{a,b}}^{2}$, and ${f_{b,b}}^{2}$, respectively. Outliers were removed from ${T_{i,a,a}}^{2}$, ${T_{i,b,a}}^{2}$, ${T_{i,a,b}}^{2}$, and ${T_{i,b,b}}^{2}$ for a robust estimation.

#### *Calculate the non-overlapping area between distributions*

The non-overlapping area of two probability distribution functions is used to describe the difference between two sets of *T*^2^ statistics.

The non-overlapping area of ${f_{a,a}}^{2},{f_{b,a}}^{2}$ is

$$D_{b,a}=\left\{\begin{array}{c} 1,if\;\;min_{a,a}>max_{b,a},or\;\;min_{b,a}>max_{a,a}\\ 1-\int_{x_{o}}^{x_{e}}\min\left\{{f_{b,a}}^{2},{f_{a,a}}^{2}\right\},if\;\;min_{a,a}<max_{b,a},or\;\;min_{b,a}<m{\mathrm{ax}}_{a,a} \end{array}\right)$$

where

$x_{o}=\max\left\{min_{a,a},\mathrm{mi}n_{b,a}\right\},\;x_{e}=\min\left\{max_{a,a},\mathrm{ma}x_{b,a}\right\}\;\max_{b,a}=\max\left\{{T_{i,b,a}}^{2},i=1,2,\cdots N_{b}\right\},\max_{a,a,L}=\max\left\{{T_{i,a,a}}^{2},i=1,2,\cdots N_{a}\right\}\;\mathrm{mi}n_{b,a}=\min\left\{{T_{i,b,a}}^{2},i=1,2,\cdots N_{b}\right\},\;\;\mathrm{mi}n_{a,a,L}=\min\;\left\{{T_{i,a,a}}^{2},i=1,2,\cdots N_{a}\right\}$

The non-overlapping area of ${f_{a,b}}^{2},{f_{b,b}}^{2}$ is

$$D_{a,b}=\left\{\begin{array}{c} 1,if\;\;min_{b,b}>\max_{a,b},or\;\;\mathrm{mi}n_{a,b}>\max_{b,b}\\ 1-\int_{x_{o}}^{x_{e}}\min\left\{{f_{a,b}}^{2},{f_{b,b}}^{2}\right\},if\;\;min_{b,b}<\max_{a,b},or\;\;\mathrm{mi}n_{a,b}<\max_{b,b} \end{array}\right)$$

where

$x_{o}=\max\left\{min_{b,b},\mathrm{mi}n_{a,b}\right\},\;x_{e}=\min\left\{max_{b,b},\mathrm{ma}x_{a,b}\right\}\;\max_{b,b}=\max\left\{{T_{i,b,b}}^{2},i=1,2,\cdots N_{b}\right\},\;\;\max_{a,b}=\max\left\{{T_{i,a,b}}^{2},i=1,2,\cdots N_{a}\right\}\;\mathrm{mi}n_{b,b}=\min\left\{{T_{i,b,b}}^{2},i=1,2,\cdots N_{b}\right\},\;\;\mathrm{mi}n_{a,b}=\min\;\left\{{T_{i,a,b}}^{2},i=1,2,\cdots N_{a}\right\}$

#### *Calculate the PM score*

The PM score is defined as $\max\;\;\{D_{a,b},D_{b,a}\}$. Compared to other non-parametric distance measures, such as Kullback–Leibler divergence, the PM score has several advantages. The profile change measure is designed under symmetry. The PM score has finite domain ranges from 0 to 1. A Kolmogorov-Smirnov test is applied to the *T*^2^ statistics to determine whether a statistically significant difference exists between conditions.

### PM2RA applications

The PM score of any defined sub-community that is referring to two or more microbes can be calculated with PM2RA. We proposed two main functions of PM2RA (Figure 1D).

#### *Constructing the RA network via 2D scanning*

Every two microbes constitute a sub-community. After traversing all sub-communities, a weighted network is built to visualize the overall RAs. In the RA network *G=*(*V, E,*) *V* is the set of nodes representing microbes and *E* is the set of edges denoting the RAs between the two conditions. The edge width and node size denote the PM score and topological degree, respectively. In this pairwise network, hub microbes, which have extensively altered associations between two compared conditions, can be identified.

#### *Module search*

In practice, the sub-communities with the largest RAs between conditions are needed to guide microbial interventions for many diseases. These sub-communities may consist of two to more microbes as the interaction between microbes is not necessarily dual. We are interested in non-redundant sub-communities with maximum PM scores (named “modules”). The modules with the largest RA between conditions are useful to describe microbial interventions for many diseases. As we can see, in a microbial community, the total number of rational sub-communities $\sum_{i=3}^{i=p}C_{p}^{i}=2^{p}-p\left(p-1\right)-p-1$ is extremely large when the number of microbes, *p*, grows. A greedy algorithm is designed to search sub-communities with large PM scores as follows:

Step 1, profile changes for all sub-communities of exactly two microbes are calculated. Step 2, *N* sub-communities with the top PM scores and no overlapping microbes are selected and marked as seed communities. Step 3, a new sub-community set is created and able to be searched. New sub-communities are generated by adding a new microbe to a seed community, whose dimension is the dimension of the seed community plus 1, and by combining two seed communities, whose dimension is twice those of the original seed communities. Step 4, the PM scores of all sub-communities newly generated in step 3 are calculated. Step 5, all the calculated PM scores resulting from steps 1 and 4 are ranked. Step 6, *N* sub-communities with the top PM scores and no overlapping microbes with each other are selected based on the data list in step 5, and marked as new seed communities. Loop into step 3 and start the iteration. Finally, when the iteration time exceeds the pre-set threshold, or the results of two iterations converge, searching is stopped and *N* microbe modules are found.

### PM2RA implementation

In the PM2RA framework, we considered several characteristics of the microbiome sequencing data. In the microbiome data-processing procedure, the most common strategy to manage zero inflation is filtering out taxa with relatively low presence, such as features present in less than 5%, 10%, or even 50% of samples [29], [30]. In our analysis pipeline, microbes detected in less than 10% of samples were removed. Also, in the application of the PM2RA web server and R script, users could set a self-defined prevalence filter to remove microbes with inflated zeros. A false discovery rate (FDR) of less than 0.05 was used as a cutoff to filter significant RAs. The average computation time of PM2RA for a dataset containing 100 features (No. of calculations: $C_{100}^{2}$) is 30 min (R with parallel computing on CentOS Linux release 7.6.1810 with E5-2680 v4, eight cores).

### PM2RA performance evaluation

Several types of datasets were downloaded to evaluate the performance of PM2RA. The 16S rRNA sequencing data for colorectal carcinoma (CRC), overweight, and obesity samples were downloaded from MicrobiomeHD [31]. The metagenomic sequencing data for CRC were downloaded from the published dataset [5]. The original dataset contained four cohorts from China, Austria, the United States (US), and France/Germany, respectively. Because samples in the US dataset were collected more than 20 years ago and had no significant RA being detected (Table S1), this dataset was excluded from the following analysis. Two diabetes datasets were downloaded from the published datasets [32].

The performance of PM2RA was compared with that of other relevant methods using artificial datasets generated based on the COMBO dataset [33]. This dataset contains operational taxonomic units (OTUs) from 100 samples. To evaluate the false positive rate (FPR) of PM2RA, we randomly separated samples into two groups with an even sample size of 50, where it is assumed that the relationship between any two OTUs does not change between the two groups. This process was repeated 100 times to generate 100 matched artificial case and control datasets. PM2RA and other methods were applied to these datasets, and the FPRs were calculated and compared. To evaluate the false negative rate (FNR) of PM2RA, we interchanged the abundances of any two OTUs each time (*e.g.*, A and B),  which were not detected to be correlated by both SparCC and SPIEC-EASI, to generate the case datasets. It is assumed that the relationship between A/B and other intact OTUs will change in such a synthetic case dataset, which is defined as the “true positive”. The control dataset is the intact one. PM2RA and other methods were applied to these datasets, and the FNRs were calculated and compared.

A two-step strategy was applied to all the synthetic datasets. First, the co-occurrence networks for each synthetic case and control datasets were constructed using the SPIEC-EASI and SparCC methods implemented in the R package SpiecEasi. The co-occurrence network was represented by a matrix consisting of 1 (correlated) and 0 (not correlated). Second, the case and control co-occurrence networks of each simulation were compared to obtain the changed association pairs. After these two steps, the sensitivity and specificity of these methods were compared with those of PM2RA.

The RAs detected by PM2RA could be used as features to discriminate between different conditions. We compared the feature extraction performance by the area under curve (AUC) between NetShift and PM2RA. The AUC was also calculated by directly using the whole microbe abundance data without feature extraction. This comparison was done by a random forest (RF) model (R 3.5.1, randomForest package, pROC package), and the one-sided *P* value of AUC was assigned by bootstrapping (*N* = 2000).

## Results

### PM2RA identified key microbes involved in human diseases

**Figure 2:**
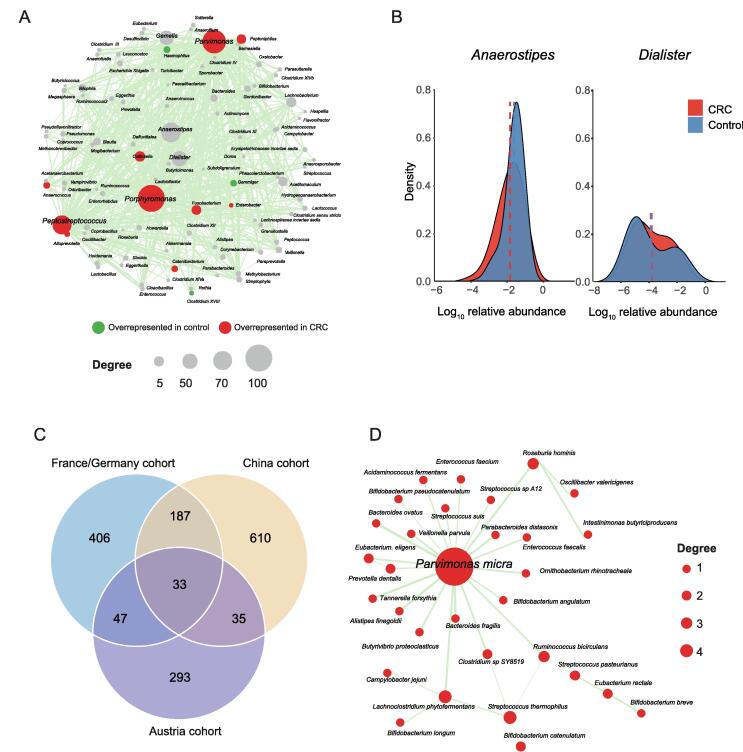
**PM2RA detected common RAs in different CRC cohorts** **A.** The RA network for a CRC cohort (case = 120, control = 172). The node color represents the abundance difference between the case and control samples: red for microbes overrepresented in the CRC samples, green for microbes overrepresented in the control, and gray fozr microbes not differentially represented. The node size is proportional to the topological degree in the network, and the edge width is proportional to the value of PM score. **B.** No abundance difference between CRC and control samples for *Anaerostipes* and *Dialister*. **C.** Thirty-three common RAs across the three CRC cohorts from Austria (case = 46, control = 63), China (case = 73, control = 92), and France/Germany (case = 88, control = 64). **D.** The common RA network among the three CRC cohorts. CRC, colorectal carcinoma.

CRC is a key example of the complex diseases associated with the dysbiosis of gut microbiota. An RA network of 97 bacterial genera and 607 significantly altered associations was built (Figure 2A) for a published CRC dataset [31]. Thirtheen genera with abundance changes were found to be involved in the RA network. The hub genera with the five largest degrees of topology were identified as *Porphyromonas*, *Parvimonas*, *Peptostreptococcus*, *Anaerostipes*, and *Dialister* (Figure 2A). Accumulating evidence has shown that *Peptostreptococcus*, *Porphyromonas,* and *Parvimonas* are overrepresented in CRC and promote the progression of oral cancer and other cancers of the upper digestive tract [34], [35]. Although the other two hub genera, *Anaerostipes* and *Dialister*, showed no difference in average abundance between the control and CRC groups (Figure 2B), their associations with many other microbes were significantly altered (Figure 2A, Figure S1). *Anaerostipes* species (*e.g.*, *Anaerostipes butyraticus*, *Anaerostipes caccae*, and *Anaerostipes hadrus*) are butyrate-producing bacterial species that play a key role in the maintenance of gut barrier functions [36]. *Dialister* is reported to be overrepresented in oral cancer [37]. Therefore, this result implicated that PM2RA can help find bacteria that affect CRC progression more accurately by searching for the RAs between microbes.

The gut microbiome is highly dynamic and can be influenced by cohort-specific noise. Thus, the results from differential abundance analysis may not be reproducible across different populations [38]. To investigate the robustness of PM2RA, we applied it to the metagenomic sequencing data of CRC patients and control subjects from Austria, China, and Franch/Germany cohorts [5] (Figure S2A–C). Thirty-three common RAs were observed across the three cohorts (Figure 2C). Consistent with the results obtained from 16S rRNA sequencing data, *Parvimonas micra* was identified as the top hub in the common RA network (Figure 2D). For example, the associations involving *Parvimonas micra* were extensively altered in the CRC group compared with the normal controls across the population. However, when measured by differential abundance, only three bacterial species were commonly detected in all three cohorts (Figure S2D), indicating that PM2RA methodology is robust in identifying RAs.

**Figure 3:**
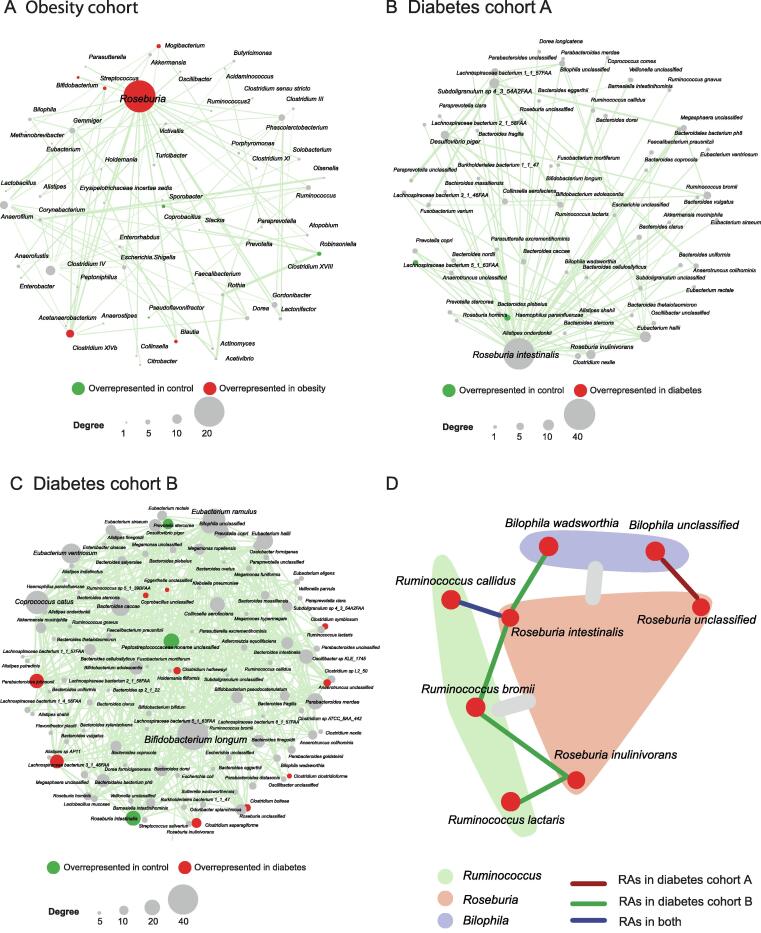
**PM2RA detected common RAs in multiple metabolic diseases** **A.** The RA network for obesity (case = 193, control = 451). **B.** and **C.** The RA networks for diabetes cohorts A (case = 57, control = 79) and B (case = 99, control = 99), respectively. **D.** The common RAs observed in obesity and diabetes datasets. The gray bars between *Roseburia* (colored as a pink module) and *Ruminococus* (colored as a green module) and between *Roseburia* and *Bilophila* (colored as a purple module) represent the common association changes observed across the obesity and diabetes datasets at the genus level. The red, green, and blue lines between species represent the association changes observed in diabetes cohort A, B, and both, respectively.

We further assessed the robustness of PM2RA by investigating whether a common RA network can be observed in related diseases. PM2RA was applied to three closely linked metabolic disorders: overweight, obesity, and diabetes. No significant RAs or differential microbes were identified in the overweight cohort [normal: body mass index (BMI) < 25; overweight: 25 < BMI < 30], indicating that BMI is not an informative index to assess a person’s disease state as has been previously reported [39]. In the obesity dataset (BMI > 30), an RA network of 85 altered associations and 97 bacterial genera was observed, with *Roseburia* having the most extensively altered associations among all the genera (Figure 3A). Moreover, in diabetes cohorts A and B [40], there were 49 and 45 association changes involving *Roseburia* spp*.*, respectively. In diabetes cohort A, *Roseburia intestinalis* dominated the RA network (Figure 3B), while in diabetes cohort B, *Bifidobacterium longum* was the top hub species (Figure 3C). The clinical information showed comparable BMIs but indicated a lower severity of dyslipidemia in diabetes cohort B. Studies have reported that *Bifidobacterium* spp. have anti-obesogenic or anti-diabetic potential [41]. The activated association changes with *Bifidobacterium longum* in diabetes cohort B may, therefore, be one explanation for the observed difference in dyslipidemia. By combining the three datasets, common association changes between *Roseburia* spp. and *Ruminococcus* spp. were identified, as well as changes between *Roseburia* spp. and *Bilophila* spp. (Figure 3D). *Roseburia* is a major butyrate-producing genus, and the modification in *Roseburia* spp. may affect various metabolic pathways [42]. In agreement with the PM2RA analysis, animal experiments have demonstrated that *Roseburia* spp. can regulate the host immune system and reduce intestinal inflammation, which is also a marker of obesity and metabolic dysfunctions [43], [44].

Taken together, the consistent results obtained in datasets using different sequencing strategies and different cohorts indicate the robustness of PM2RA in identifying RA networks in various diseases.

### HD PM2RA analysis complements to 2D scanning

**Figure 4:**
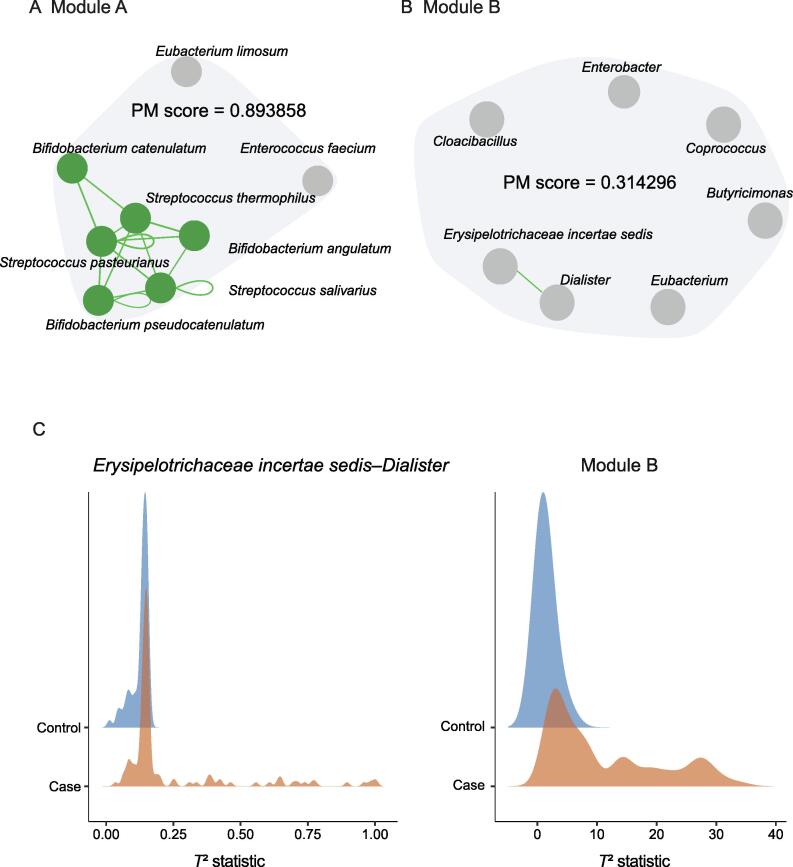
**The HD PM2RA analysis is complementary to 2D scanning** **A.** and **B.** Examples of HD RAs. The lines between microbes represent the RAs detected with 2D scanning, and the PM score denotes the RA value for the module consisting of the presented microbes. **C.** The distribution of the *T*^2^ statistics for 2D scanning (*Erysipelotrichaceae incertae sedis* and *Dialister*) and HD module shown in (B) in the case and control samples. The non-overlapping area (PM score) is larger in the HD module.

Associations between microbes are not necessarily structured in a paired way, and multiple microbes can form closely interacting sub-communities. The ability of PM2RA to quantify RAs involving multiple microbes makes it applicable to identifying RAs in such communities. We, therefore, tested the performance of PM2RA in HD microbial communities in the datasets mentioned above. By applying the greedy algorithm, HD RAs (FDR < 0.05, PM score > 0.6) were identified in all datasets except for the obesity and overweight datasets (Table S2). Most modules contained more than two microbes, indicating potential associations among multiple bacteria. Furthermore, many HD RAs contained microbe pairs that were not significantly altered at the 2D level (Figure 4A and B), illustrating the great ability of PM2RA to detect weak change signals in HD microbial communities under different conditions, which have usually been ignored by 2D scanning (Figure 4C). These results suggested that PM2RA is a promising method to quantify 2D and HD microbial RAs.

### PM2RA outperforms other methods in the RA network inference

**Figure 5:**
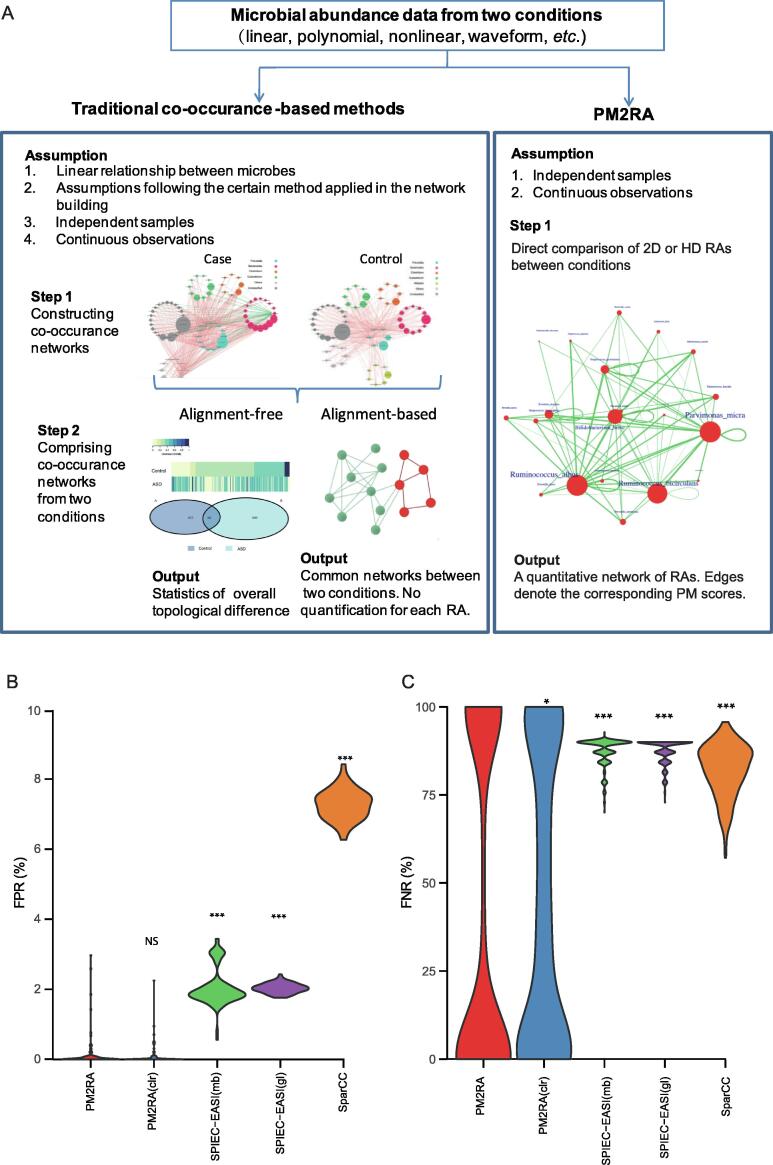
**Comparisons between PM2RA and the co-occurrence-based methods** **A.** The difference and advantage of PM2RA comparing to traditional co-occurrence-based methods. **B.** and **C.** Comparison of the FPRs (B) and FNRs (C) of different methods in detecting RAs. The difference was compared between PM2RA and other methods using the Mann–Whitney *U* test. *, *P* < 0.05; **, *P* < 0.01; ***, *P* < 0.001; NS, not significant. FPR, false positive rate; FNR, false negative rate.

In a traditional workflow (Figure 5A, left panel), the microbial co-occurrence networks are constructed from the pairwise correlation, inverse covariance, or other statistics based on the microbial abundances in the case and control samples, respectively. The networks are then further compared by alignment-based or alignment-free methods. There are three drawbacks inherent in this pipeline. First, it is based on the pairwise correlation network, but it is unclear whether the correlation is a proper measure of association. Second, the association of microbiota is not necessarily dual. For example, multiple bacteria could form a tight community with weaker associations between any two members within it. Thus, the pairwise relationship analysis might ignore some functional associations consisting of multiple microbes. Third, this comparison can neither quantify the association changes between conditions nor quantify the degree of association changes. But rather, as shown in Figure 5A (right panel), PM2RA directly compares the RA(s) among two or more microbes between conditions, does not need to build a co-occurrence network like that of the traditional methods, and quantifies the RA as a PM score.

To evaluate the performance of PM2RA with realistic synthetic microbiome data, we generated the artificial datasets based on a real microbiome dataset (see the Method section for details). PM2RA and the other two methods, which were widely used to infer co-occurrence networks (*i.e.*, SPIEC-EASI [45] and SparCC [46]), were applied to these datasets. The average FPR of PM2RA was significantly lower than those of the co-occurrence-based methods [PM2RA: 0.1%; SPIEC-EASI (mb-based): 2.1%; SPIEC-EASI (glasso-based): 2.0%; SparCC: 7.3%] (Figure 5B; Table S3), indicating its high specificity. Additionally, PM2RA showed a significantly lower FNR than the co-occurrence-based strategies [PM2RA: 33.5%; SPIEC-EASI (mb-based): 87.6%; SPIEC-EASI (glasso-based): 87.3%; SparCC: 82.1%] (Figure 5C; Table S4). The FNR of PM2RA is dumbbell-shaped (Figure 5C), suggesting that it is affected by the effectiveness of the case datasets, and is sensitive to the correlations missed by SPIEC-EASI and SparCC (see the Discussion section for details).

The compositional data is widely used in microbiome data analysis. However, it has been proposed that it could produce superior results in correlation analysis [47]. Therefore, to test the effect of compositional data on PM2RA performance, a centered-log-ratio (clr) transformation [47] was applied to the abovementioned artificial datasets. A similar FPR (*P* = 0.11) was observed when applying PM2RA to the compositional and clr-transformed data (Figure 5B). However, the FNR of PM2RA on the compositional data was significantly lower than that of the clr-transformed data (33.5% *vs.* 43.4%; *P* = 3.852E–08) (Figure 5C). Taken together, the analysis showed that the compositional data were preferred in PM2RA to the clr-transformed data.

NetShift is a co-occurrence-based method developed to quantify rewiring and community changes in microbial association networks between health and disease states [48]. It was designed to produce a score that identifies important microbial taxa that serve as “drivers” from the first state to the second. NetShift was applied to the datasets of CRC (Figure S3A–D) and metabolism disorders (Figure S4A–D) as mentioned above. Two common driver species were identified across the three CRC cohorts from Austria, China, and France/Germany (Figure S3E), that is *Butyrivibrio proteoclasticus* and *Streptococcus pyogenes*. However, the previously identified microbes involved in the disease, such as *Bacteroides fragilis*, *Fusobacterium nucleatum*, *Porphyromonasa saccharolytica*, *Parvimonas micra*, *Prevotella intermedia*, *Alistipes finegoldii*, and *Thermanaerovibrio acidaminovorans* [5]*,* were not captured. On the other hand, five out of the seven previously identified species were commonly detected across three CRC datasets by PM2RA (Figure 2D). More than 40% of the NetShift-identified drivers were shared by the obesity and overweight samples (Figure S4E), and four drivers were shared by the two diabetes datasets (Figure S4F), *i.e.*, *Alistipesshahii*, *Anaerotruncus colihominis*, *Eubacterium hallii*, and *Eubacterium ventriosum*. However, few of these drivers are associated with metabolism disorders. For example, the oft-reported species, *Ruminococcus.* spp*.* and *Roseburia.* spp., were detected by PM2RA (Figure 3D) but not recognized as drivers by NetShift.

### PM2RA is a good feature extraction tool in distinguishing case and control samples

**Figure 6:**
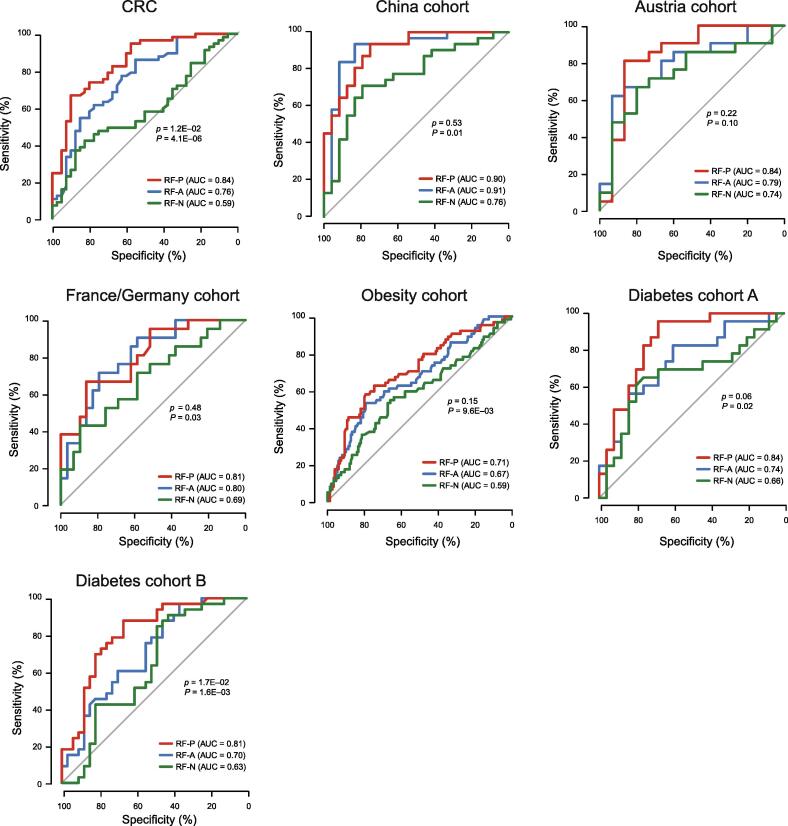
**The RF model of PM2RA distinguished case and control samples** *p* and *P* denote the *P* values for comparison between Hotelling’s *T*^2^ statistics (RF-P) with total microbe abundance (RF-A) and microbe abundance of drivers detected by NetShift (RF-N), respectively. RF, random forest.

To test whether the microbial relationship represented in PM2RA (by Hotelling’s *T*^2^ statistics) captured important information that distinguished case samples from control samples, we generated RF models using multiple types of inputs from the abovementioned datasets: the total microbe abundance (RF-A), microbe abundance of drivers detected by NetShift (RF-N), and the Hotelling’s *T*^2^ statistics of paired microbes (RF-P). The RF-P model achieved higher AUC values on the ROC curves than the RF-A model in two of the seven datasets (Figure 6). In the comparison of RF-P and RF-N models, significantly higher AUC values were observed in the six of the seven datasets (Figure 6), indicating that the RA revealed more information than the abundance shift of drivers identified by the co-occurrence-based method in the pathogenesis of these diseases. Besides, the hub microbes in the RA network were highly overlapped with the microbes with the highest importance scores in the RF-A model (Figures S5 and S6). In the 16S rRNA CRC dataset, the top three hub microbes (*i.e.*, *Porphyromonas*, *Parvimonas*, and *Peptostreptococcus*) (Figure 2A) were ranked as three of the top four important features in the RF-A model (Figure S5A). *Parvimonas micra*, the most notable hub microbe commonly detected in multiple CRC datasets (Figure 2D), was among the top five most important species in three CRC cohorts from Austria, China, and France/Germany (Figure S5B–D). The *Roseburia* and *Bilophila* species, which were commonly detected in obesity and diabetes cohorts by PM2RA (Figure 3D), were identified by the RF-A model as top features (Figure S6A–C). However, the *Ruminococcus* species identified by PM2RA was not recognized as a top feature in the RF-A model, which may represent the additional information captured by PM2RA that contributed to its higher classification power. These results suggested that the Hotelling’s *T*^2^ statistic transformation in PM2RA not only preserves the most important feature that distinguishes health and disease statues but also provides extra information underlying the pathogenesis of human diseases.

## Discussion

Microbial association analysis is an important complement to the differential abundance analysis in the study of gut microbiota dysbiosis in diseases. In the current study, we developed an innovative analysis method to detect and quantify microbial RAs. PM2RA measures the RAs of microbial sub-communities, without initially constructing a co-occurrence network for each condition. We demonstrated that PM2RA has higher sensitivity and specificity than the traditional co-occurrence-based methods. The RF analysis revealed that the microbial RA represented by PM2RA distinguishes disease and health statuses more robustly than the abundance shift of driver microbes identified by co-occurrence-based methods. Furthermore, the applications of PM2RA in several disease datasets demonstrate the robustness of PM2RA.

In our applications, PM2RA showed biological-reproducible results in datasets with sample size ranging from tens to hundreds. However, since PM2RA calculates RAs based on the projection distributions, the larger the sample size, the more precise the distribution estimation. We recommend applying it to datasets with more than 30 samples for each of the compared conditions. It is hard to define the true positive RA when evaluating the sensitivity of PM2RA, due to the lack of statistical methods to define and quantify RAs. We used SPIEC-EASI and SparCC to define “uncorrelated” microbes and interchanged the abundances of any two uncorrelated microbes to generate the case datasets. However, some types of correlations can still be neglected, thus rendering the exchange not fully effective. Therefore, the results might have shown an underestimated sensitivity. The FNR of PM2RA is dumbbell-shaped (Figure 5C), suggesting that the FNR of PM2RA is affected by the effectiveness of the case datasets. A low FNR will be observed when the exchanged OTUs are independent of each other; otherwise, PM2RA recognizes the relationships between them and most other species as similar, resulting in a high FNR. These results also indicated that PM2RA is sensitive to the correlations missed by SparCC and SPIEC-EASI.

The abundance of microbial OTUs from amplicon-based datasets is usually compositional, where counts are normalized to the total number of counts in the sample. Applying traditional correlation analysis to such data may produce spurious results [47]. Because PM2RA detects RAs without constructing co-occurrence networks, the influence of compositional data on its results is small. Therefore, a comparable specificity was observed when applying PM2RA to the compositional data and clr-transformed data of the synthetic datasets. However, the sensitivity of PM2RA with the clr-transformed data was significantly lower than that with the compositional data. This result might be due to the alteration of the abundance baseline caused by transformation  and the subsequent impact on the relationships inherited by the raw abundance data.

Notably, we considered no environmental factors that could lead to possible overdispersion of the microbiome data in PM2RA. Since the purpose of PM2RA is to compare relationships of microbes between two conditions, generally, given the experimental designs of most of the case-control studies, the samples sequenced in each condition were similar in other factors, such as distributions of age and gender and environmental factors, except for the designed factor. Therefore, in the detection of RAs, the effects of other factors on the differential correlation between two conditions were ignored. Mathematically, we simply removed the outliers when calculating the PM scores. However, considering the overdispersion caused by the environmental factors that might be ignored or not well-balanced in the experimental designs may be a way to improve the performance of PM2RA further.

In conclusion, PM2RA is a novel method for identifying and directly quantifying RAs in microbial communities. It circumvents the drawbacks of the co-occurrence-based methods. Applying PM2RA to multiple human diseases reveals biologically significant results. The ability of PM2RA to detect community-level dysbiosis may make PM2RA a useful tool for exploring the functional alterations of microbes as a whole in a variety of diseases or biological conditions, to provide additional hints about the pathogenesis of human diseases.

## Code availability

The source code of PM2RA and additional codes and datasets used in this work are available at https://github.com/Xingyinliu-Lab/PM2RA. A web-based PM2RA service is available at http://www.pm2ra-xingyinliulab.cn/.

## CRediT author statement

**Zhi Liu:** Investigation, Validation, Formal analysis, Writing - original draft, Writing - review & editing. **Kai Mi:** Conceptualization, Methodology, Software, Investigation, Writing - original draft, Writing - review & editing. **Zhenjiang Zech Xu:** Validation, Writing - review & editing. **Qiankun Zhang:** Validation. **Xingyin Liu:** Conceptualization, Project administration, Investigation, Writing - review & editing, Supervision, Validation, Funding acquisition. All authors read and approved the final manuscript.

## Competing interests

The authors declare that they have no conflict of interest.

## Supplementary material

Supplementary Figure S1 **Relationship alterations (RAs) involved by two microbes without abundance change**. The RAs involved by Dialister (**A**) and Anaerostipes (**B**).

Supplementary Figure S2 **The relationship alteration (RA) network for multiple CRC cohorts**. The RA network for cohorts from France/Germany (**A**) (case = 88, control = 64), China (**B**) (case = 73, control = 92), and Austria (**C**) (case = 46, control = 63). The node color represents the abundance difference between the case and control samples; red for microbes overrepresented in the CRC samples, green for microbes overrepresented in the control and gray for microbes not differentially represented. The size of nodes is proportional to their degree in the network, and the width of edges is proportional to the value of PM score. **D**. The overlap of differential represented microbes across three CRC cohorts.

Supplementary Figure S3 **The drives identified by “Netshift” method in colorectal carcinoma cohorts**. The driver genera idetified by “Netshift” in CRC cohorts from Reference [31] (**A**) (n = 120, control = 172), Austria (**B**) (case = 46, control = 63), China (**C**) (case = 73, control = 92), and France/Germany (**D**) (case = 88, control = 64). **E**. The overlap of drives across three metagenomic sequenced CRC cohorts.

Supplementary Figure S4 **The drives identified by “Netshift” method in metabolism disorders**. The driver genera identified by “Netshift” in metabolism disorder cohorts of overweight (**A**), Obesity (**B**), Type 2 diabetes dataset A (**C**), and Type 2 diabetes dataset B (**D**). E.The overlap of drives between over-weight and obesity. **F**. The overlap of drives between two diabetes datasets.

Supplementary Figure S5 **The important features extracted by RF model generated on the microbe abundance in CRC cohorts**. The top 20 features extracted by the random forest model based on the microbe abundance in the 16s rRNA sequenced CRC cohort (**A**), China (**B**), Austria (**C**) and France/Germany cohort (**D**). The red labeled microbes are taxa identified by PM2RA as hubs of the RA network.

Supplementary Figure S6 **The important features extracted by RF model generated on the microbe abundance in obesity and diabetes cohorts**. The top 20 important features by the random forest model generated on the microbe abundancein the obesity cohort (**A**), Type 2 diabetes dataset A (**B**), and Type 2 diabetes dataset B (**C**). The red labeled microbes are taxa identified by PM2RA as hubs of the RA network.

## References

[1] Lynch S.V., Pedersen O. (2016). The human intestinal microbiome in health and disease. N Engl J Med.

[2] Young V.B. (2017). The role of the microbiome in human health and disease: an introduction for clinicians. BMJ.

[3] Goodrich J.K., Waters J.L., Poole A.C., Sutter J.L., Koren O., Ran B. (2014). Human genetics shape the gut microbiome. Cell.

[4] Chng K.R., Tay A.S.L., Li C., Ng A.H.Q., Wang J., Suri B.K. (2016). Whole metagenome profiling reveals skin microbiome-dependent susceptibility to atopic dermatitis flare. Nat Microbiol.

[5] Dai Z., Coker O.O., Nakatsu G., Wu W.K.K., Zhao L., Chen Z. (2018). Multi-cohort analysis of colorectal cancer metagenome identified altered bacteria across populations and universal bacterial markers. Microbiome.

[6] Mima K., Nishihara R., Qian Z.R., Cao Y., Sukawa Y., Nowak J.A. (2016). *Fusobacterium nucleatum* in colorectal carcinoma tissue and patient prognosis. Gut.

[7] Flint H.J., Duncan S.H., Scott K.P., Louis P. (2007). Interactions and competition within the microbial community of the human colon: links between diet and health. Environ Microbiol.

[8] Faust K., Raes J. (2012). Microbial interactions: from networks to models. Nat Rev Microbiol.

[9] Yadav D., Ghosh T.S., Mande S.S. (2016). Global investigation of composition and interaction networks in gut microbiomes of individuals belonging to diverse geographies and age-groups. Gut Pathog.

[10] Belenguer A., Duncan S.H., Calder A.G., Holtrop G., Louis P., Lobley G.E. (2006). Two routes of metabolic cross-feeding between *Bifidobacterium adolescentis* and butyrate-producing anaerobes from the human gut. Appl Environ Microbiol.

[11] Ramsay J., Everitt BS, Howell DC (2005). Encyclopedia of statistics in behavioral science.

[12] Ganz H.H., Doroud L., Firl A.J., Hird S.M., Eisen J.A., Boyce W.M. (2017). Community-level differences in the microbiome of healthy wild mallards and those infected by influenza A viruses. mSystems.

[13] Poudel R., Jumpponen A., Schlatter D.C., Paulitz T.C., Gardener B.B.M., Kinkel L.L. (2016). Microbiome networks: a systems framework for identifying candidate microbial assemblages for disease management. Phytopathology.

[14] Wang J., Gao Y., Zhao F. (2016). Phage-bacteria interaction network in human oral microbiome. Environ Microbiol.

[15] Faisal F.E., Zhao H., Milenkovic T. (2015). Global network alignment in the context of aging. IEEE/ACM Trans Comput Biol Bioinform.

[16] Kelley B.P., Sharan R., Karp R.M., Sittler T., Root D.E., Stockwell B.R. (2003). Conserved pathways within bacteria and yeast as revealed by global protein network alignment. Proc Natl Acad Sci U S A.

[17] Neyshabur B., Khadem A., Hashemifar S., Arab S.S. (2013). NETAL: a new graph-based method for global alignment of protein-protein interaction networks. Bioinformatics.

[18] Liao C.S., Lu K., Baym M., Singh R., Berger B. (2009). IsoRankN: spectral methods for global alignment of multiple protein networks. Bioinformatics.

[19] Yaveroğlu Ö.N., Malod-Dognin N., Davis D., Levnajic Z., Janjic V., Karapandza R. (2014). Revealing the hidden language of complex networks. Sci Rep.

[20] Milo R., Itzkovitz S., Kashtan N., Levitt R., Shen-Orr S., Ayzenshtat I. (2004). Superfamilies of evolved and designed networks. Science.

[21] Wang J., Zheng J., Shi W., Du N., Xu X., Zhang Y. (2018). Dysbiosis of maternal and neonatal microbiota associated with gestational diabetes mellitus. Gut.

[22] Woodall W.H. (2005). Introduction to statistical quality control, fifth edition. J Quality Technol.

[23] Mason R.L., Young J.C. (2002). Multivariate statistical process control with industrial applications. Society for Industrial and Applied Mathematics.

[24] Clooney A.G., Sutton T.D.S., Shkoporov A.N., Holohan R.K., Daly K.M., O’Regan O. (2019). Whole-virome analysis sheds light on viral dark matter in inflammatory bowel disease. Cell Host Microbe.

[25] Xiong M., Zhao J., Boerwinkle E. (2002). Generalized *T*^*2*^ test for genome association studies. Am J Hum Genet.

[26] Harold H. (1993). The generalization of Student’s ratio. Ann Math Stat.

[27] Chen J., King E., Deek R., Wei Z., Yu Y., Grill D. (2018). An omnibus test for differential distribution analysis of microbiome sequencing data. Bioinformatics.

[28] David S. (2015). Multivariate density estimation: theory, practice, and visualization.

[29] Hjelmsø M.H., Shah S.A., Thorsen J., Rasmussen M., Vestergaard G., Mortensen M.S. (2020). Prenatal dietary supplements influence the infant airway microbiota in a randomized factorial clinical trial. Nat Commun.

[30] Flemer B., Warren R.D., Barrett M.P., Cisek K., Das A., Jeffery I.B. (2018). The oral microbiota in colorectal cancer is distinctive and predictive. Gut.

[31] Duvallet C., Gibbons S.M., Gurry T., Irizarry R.A., Alm E.J. (2017). Meta-analysis of gut microbiome studies identifies disease-specific and shared responses. Nat Commun.

[32] Pasolli E, Truong DT, Malik F, Waldron L, Segata N. Machine learning meta-analysis of large metagenomic datasets: tools and biological insights. PLoS Comput Biol 2016;12:e1004977. 10.1371/journal.pcbi.1004977 PMC4939962 27400279

[33] Wu G.D., Chen J., Hoffmann C., Bittinger K., Chen Y.Y., Keilbaugh S.A. (2011). Linking long-term dietary patterns with gut microbial enterotypes. Science.

[34] Gholizadeh P., Eslami H., Yousefi M., Asgharzadeh M., Aghazadeh M., Kafil H.S. (2016). Role of oral microbiome on oral cancers, a review. Biomed Pharmacother.

[35] Yuan X., Liu Y., Kong J., Gu B., Qi Y., Wang X. (2017). Different frequencies of *Porphyromonas gingivalis* infection in cancers of the upper digestive tract. Cancer Lett.

[36] Rivière A, Selak M, Lantin D, Leroy F, De Vuyst L. Bifidobacteria and butyrate-producing colon bacteria: importance and strategies for their stimulation in the human gut. Front Microbiol 2016;7:979. 10.3389/fmicb.2016.00979 PMC4923077 27446020

[37] Zhao H., Chu M., Huang Z., Yang X., Ran S., Hu B. (2017). Variations in oral microbiota associated with oral cancer. Sci Rep.

[38] He Y., Wu W., Zheng H.M., Li P., McDonald D., Sheng H.F. (2018). Regional variation limits applications of healthy gut microbiome reference ranges and disease models. Nat Med.

[39] Kennedy A.P., Shea J.L., Sun G. (2009). Comparison of the classification of obesity by BMI *vs.* dual-energy X-ray absorptiometry in the Newfoundland population. Obesity.

[40] Qin J., Li Y., Cai Z., Li S., Zhu J., Zhang F. (2012). A metagenome-wide association study of gut microbiota in type 2 diabetes. Nature.

[41] Woting A., Blaut M. (2016). The intestinal microbiota in metabolic disease. Nutrients.

[42] Tamanai-Shacoori Z., Smida I., Bousarghin L., Loreal O., Meuric V., Fong S.B. (2017). *Roseburia* spp.: a marker of health?. Future Microbiol.

[43] Zhu C., Song K., Shen Z., Quan Y., Tan B., Luo W. (2018). *Roseburia intestinalis* inhibits interleukin-17 excretion and promotes regulatory T cells differentiation in colitis. Mol Med Rep.

[44] Patterson A.M., Mulder I.E., Travis A.J., Lan A., Cerf-Bensussan N., Gaboriau-Routhiau V. (2017). Human gut symbiont *Roseburia hominis* promotes and regulates innate immunity. Front Immunol.

[45] Kurtz Z.D., Müller C.L., Miraldi E.R., Littman D.R., Blaser M.J., Bonneau R.A. (2015). Sparse and compositionally robust inference of microbial ecological networks. PLoS Comput Biol.

[46] Friedman J., Alm E.J. (2012). Inferring correlation networks from genomic survey data. PLoS Comput Biol.

[47] Aitchison J. (1994). A concise guide to compositional data analysis. Lecture Notes-Monograph Series.

[48] Kuntal B.K., Chandrakar P., Sadhu S., Mande S.S. (2018). ‘NetShift’: a methodology for understanding ‘driver microbes’ from healthy and disease microbiome datasets. ISME J.

