# Supplementary figures and images for: PM2RA: A Framework for Detecting and Quantifying Relationship Alterations in Microbial Community
Source: Genomics Proteomics Bioinformatics. 2021 Feb 11;19(1):154–67. doi: 10.1016/j.gpb.2020.07.005 (PMC8498968; doi:10.1016/j.gpb.2020.07.005)

A France/Germany cohort

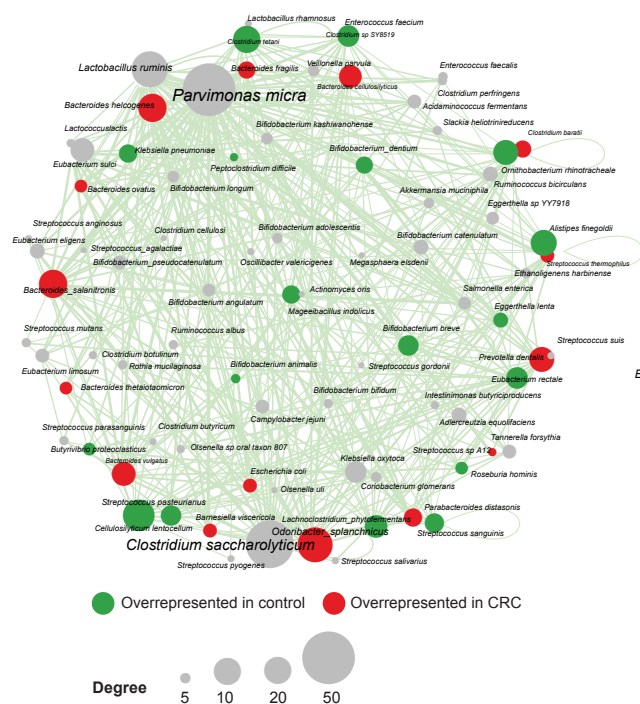

B China cohort

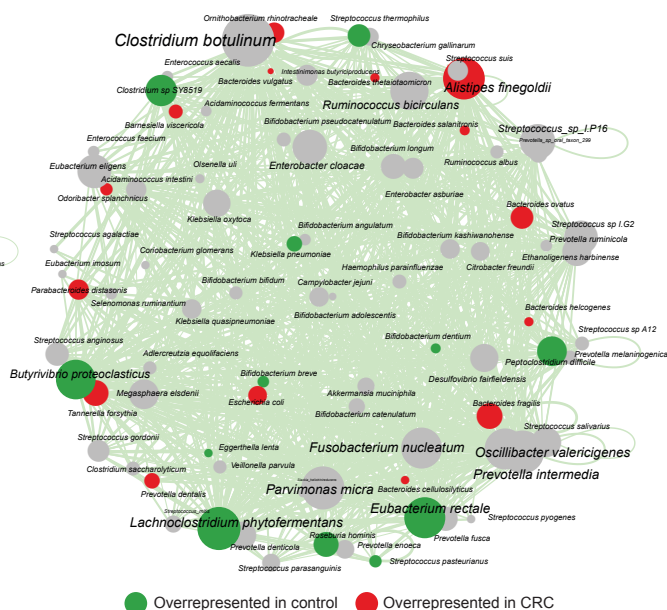

C Austria cohort

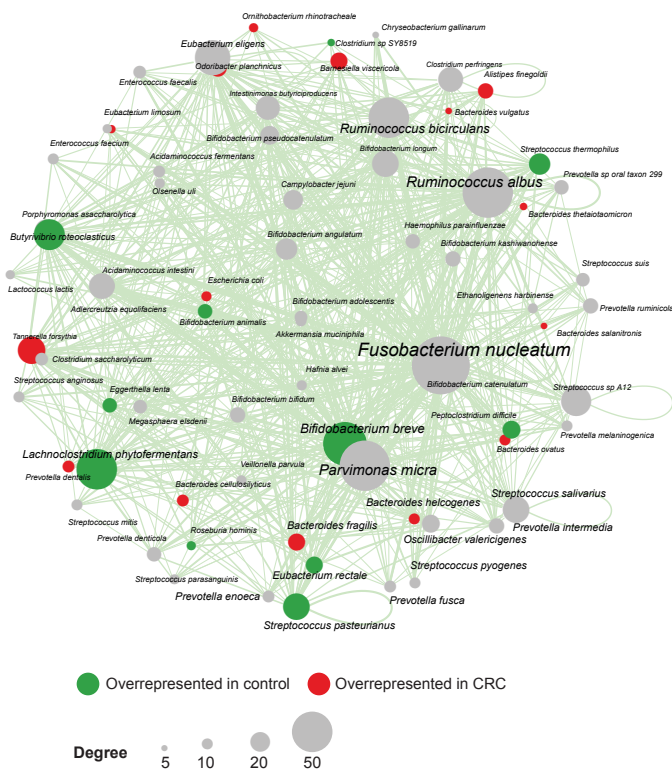

D

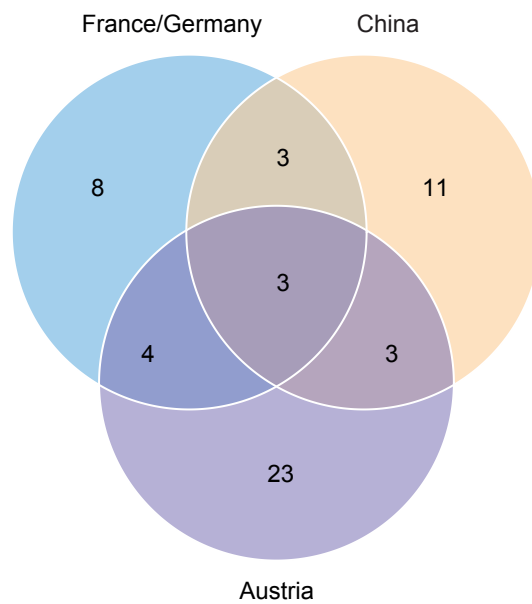

Supplement: Supplementary Figure S2 — The relationship alteration (RA) network for multiple CRC cohorts. The RA network for cohorts from France/Germany (A) (case = 88, control = 64), China (B) (case = 73, control = 92), and Austria (C) (case = 46, control = 63). The node color represents the abundance difference between the case and control samples; red for microbes overrepresented in the CRC samples, green for microbes overrepresented in the control and gray for microbes not differentially represented. The size of nodes is proportional to their degree in the network, and the width of edges is proportional to the value of PM score. D. The overlap of differential represented microbes across three CRC cohorts. [file mmc2.pdf]

## A CRC

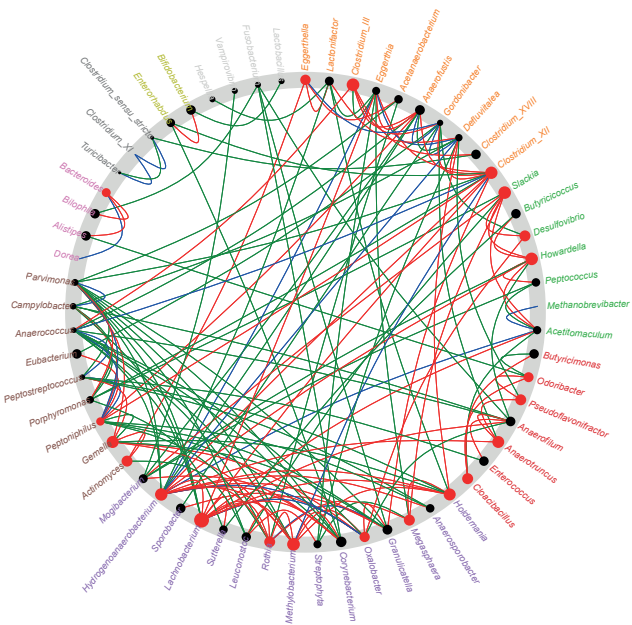

### B Austria cohort

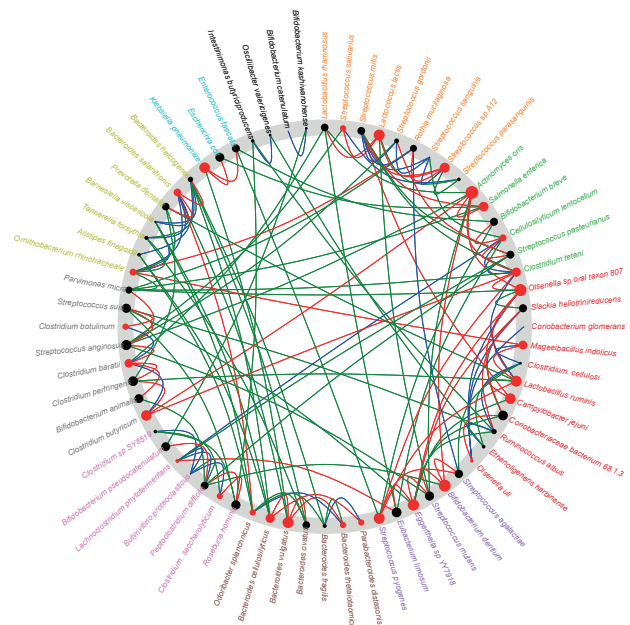

### C China cohort

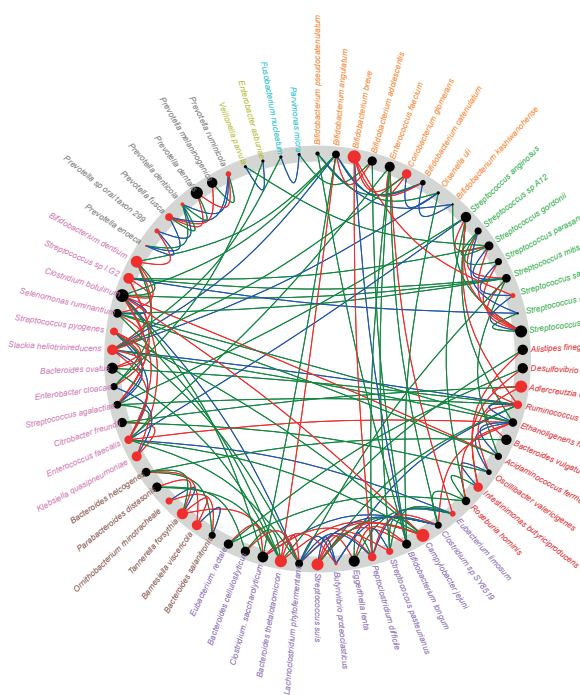

#### D France/Germany cohort

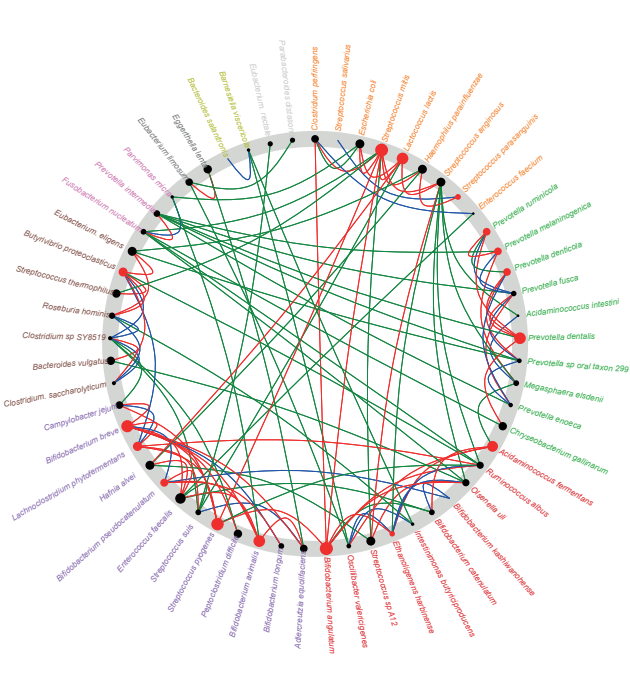

E

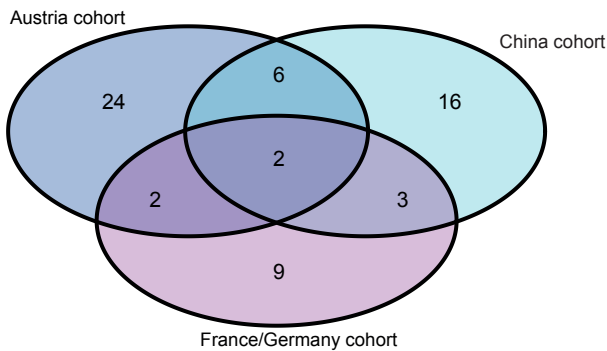

Supplement: Supplementary Figure S3 — The drives identified by “Netshift” method in colorectal carcinoma cohorts. The driver genera idetified by “Netshift” in CRC cohorts from Reference [31] (A) (n = 120, control = 172), Austria (B) (case = 46, control = 63), China (C) (case = 73, control = 92), and France/Germany (D) (case = 88, control = 64). E. The overlap of drives across three metagenomic sequenced CRC cohorts. [file mmc3.pdf]

A Overweight

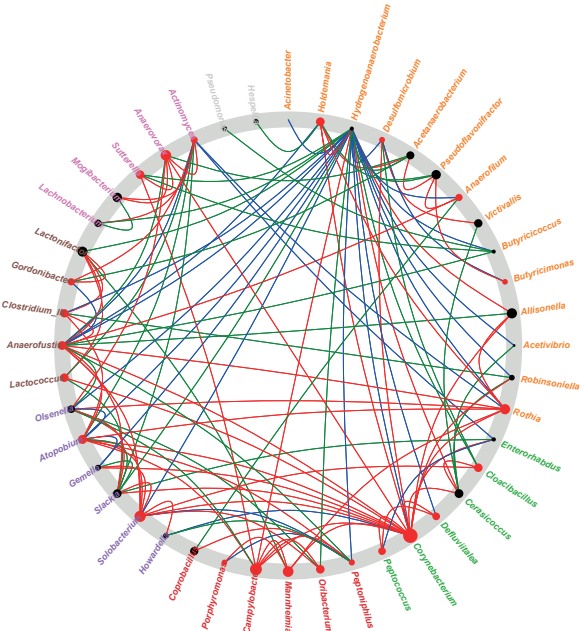

C Diabetes cohort A

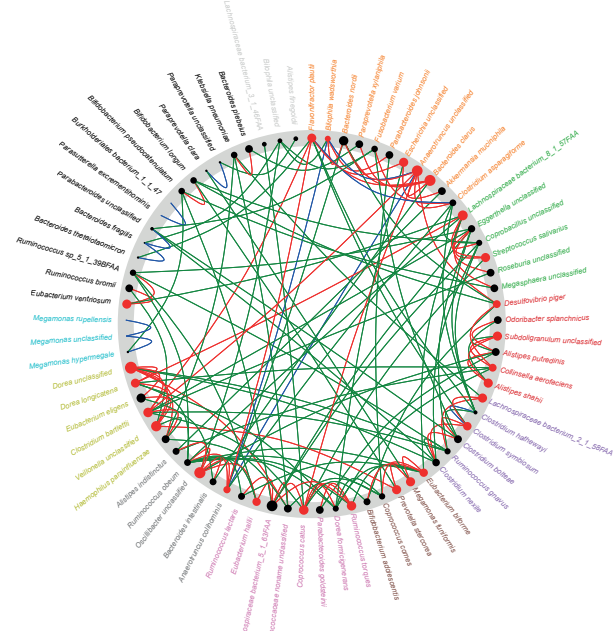

B Obesity

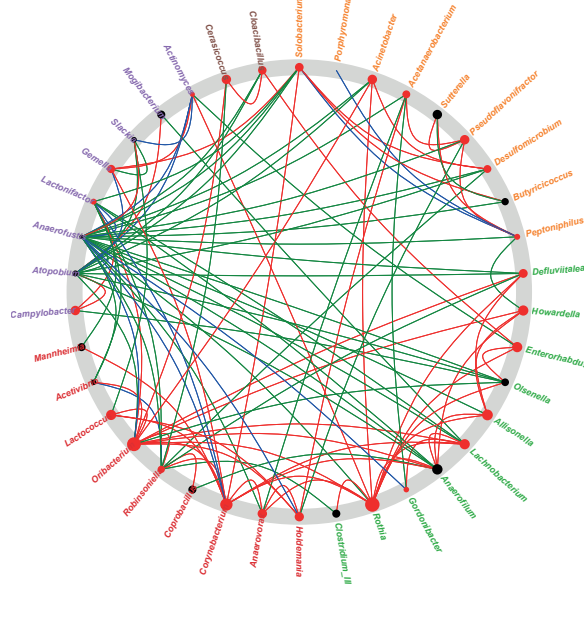

D Diabetes cohort B

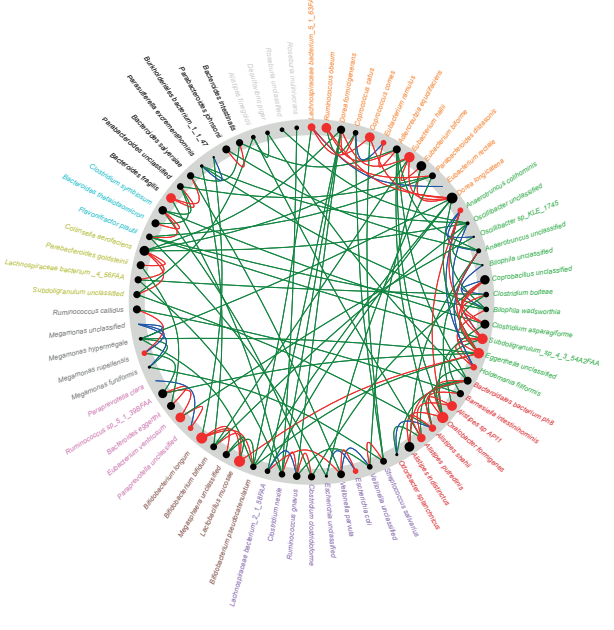

E

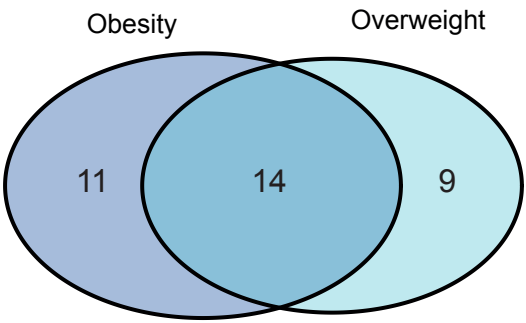

F

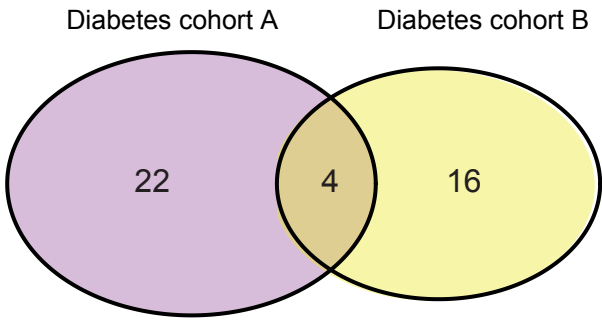

Supplement: Supplementary Figure S4 — The drives identified by “Netshift” method in metabolism disorders. The driver genera identified by “Netshift” in metabolism disorder cohorts of overweight (A), Obesity (B), Type 2 diabetes dataset A (C), and Type 2 diabetes dataset B (D). E.The overlap of drives between over-weight and obesity. F. The overlap of drives between two diabetes datasets. [file mmc4.pdf]

A CRC

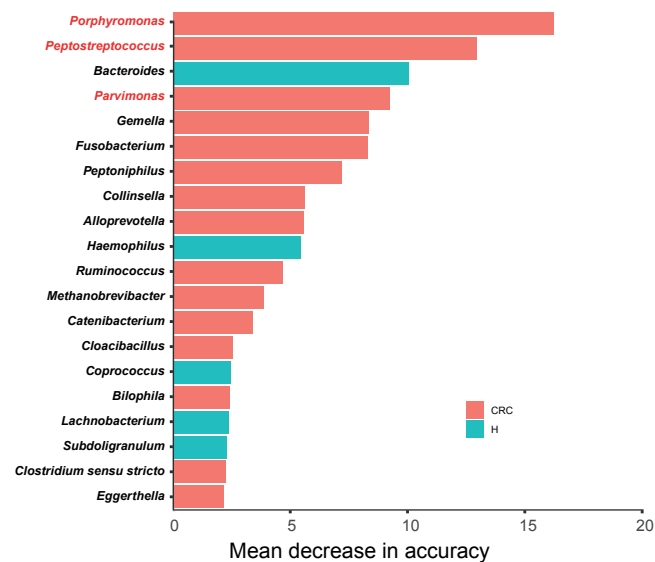

B China corhot

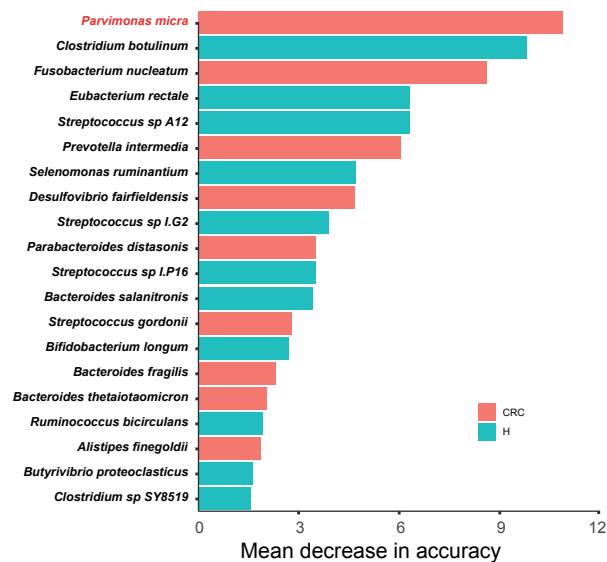

C Austria corhot

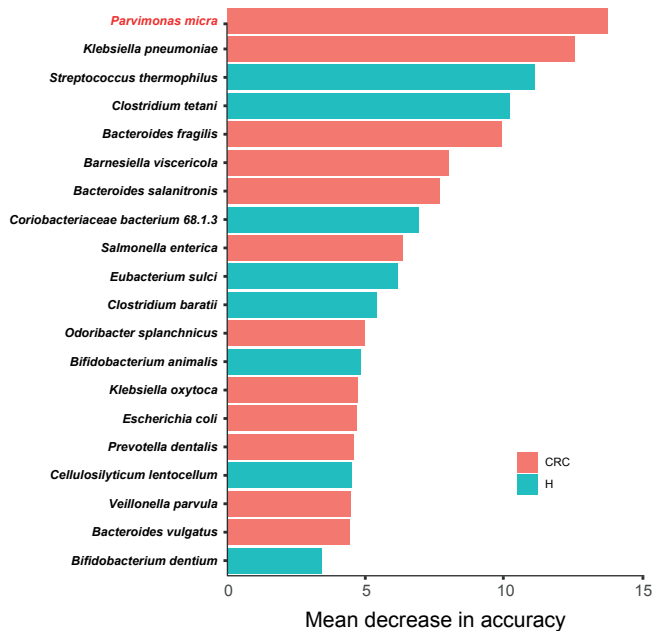

D France/Germany corhot

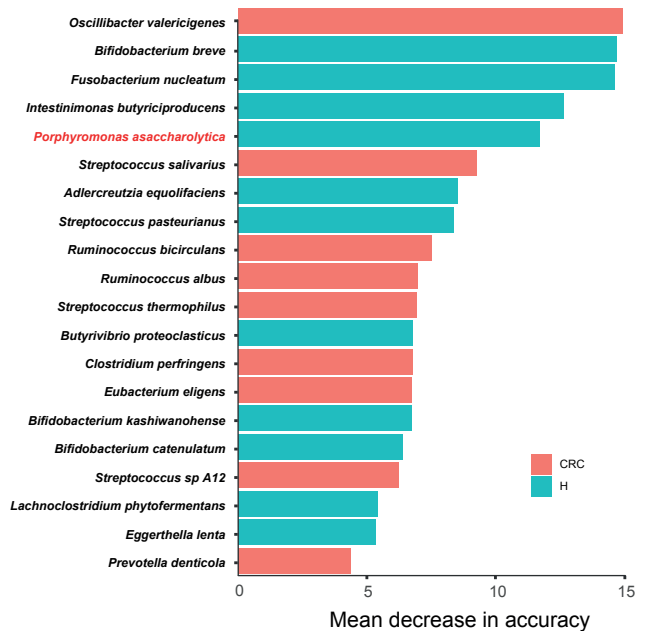

Supplement: Supplementary Figure S5 — The important features extracted by RF model generated on the microbe abundance in CRC cohorts. The top 20 features extracted by the random forest model based on the microbe abundance in the 16s rRNA sequenced CRC cohort (A), China (B), Austria (C) and France/Germany cohort (D). The red labeled microbes are taxa identified by PM2RA as hubs of the RA network. [file mmc5.pdf]

A Obesity

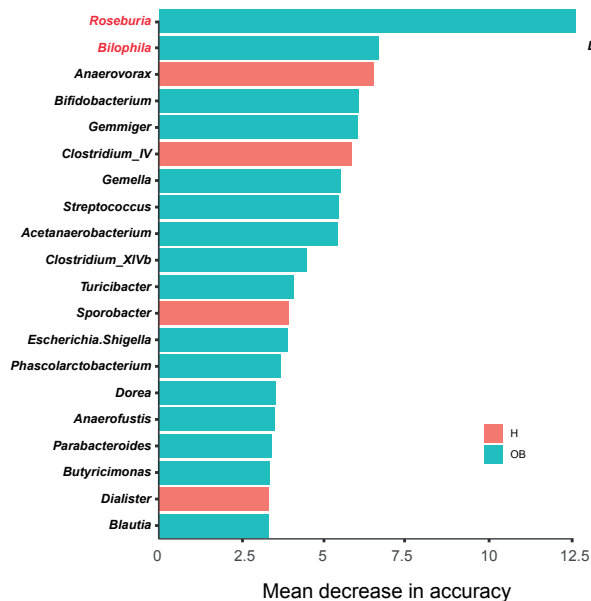

B Diabetes cohort A

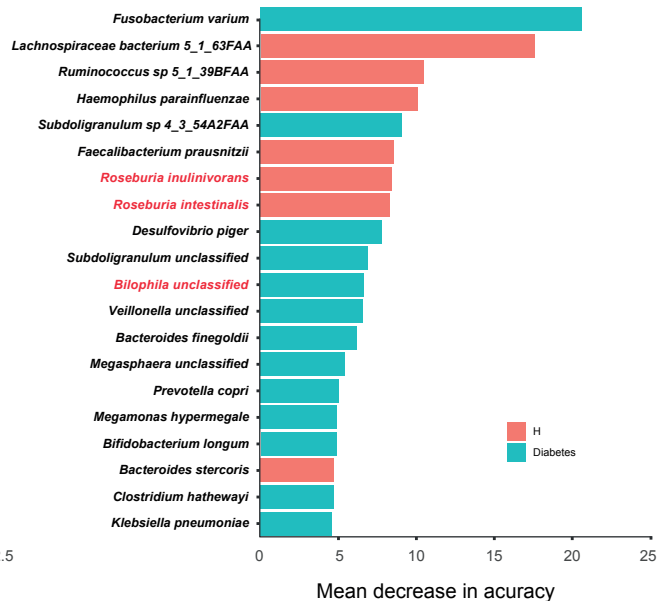

C Diabetes cohort B

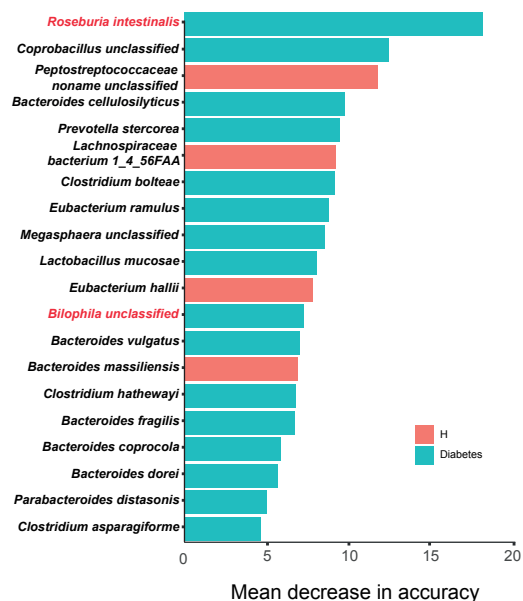

Supplement: Supplementary Figure S6 — The important features extracted by RF model generated on the microbe abundance in obesity and diabetes cohorts. The top 20 important features by the random forest model generated on the microbe abundancein the obesity cohort (A), Type 2 diabetes dataset A (B), and Type 2 diabetes dataset B (C). The red labeled microbes are taxa identified by PM2RA as hubs of the RA network. [file mmc6.pdf]
