# Supplementary material for: PM2RA: A Framework for Detecting and Quantifying Relationship Alterations in Microbial Community
Source: Genomics Proteomics Bioinformatics. 2021 Feb 11;19(1):154–67. doi: 10.1016/j.gpb.2020.07.005 (PMC8498968; doi:10.1016/j.gpb.2020.07.005)

A RA of Dialister

B RA of Anaerostipes

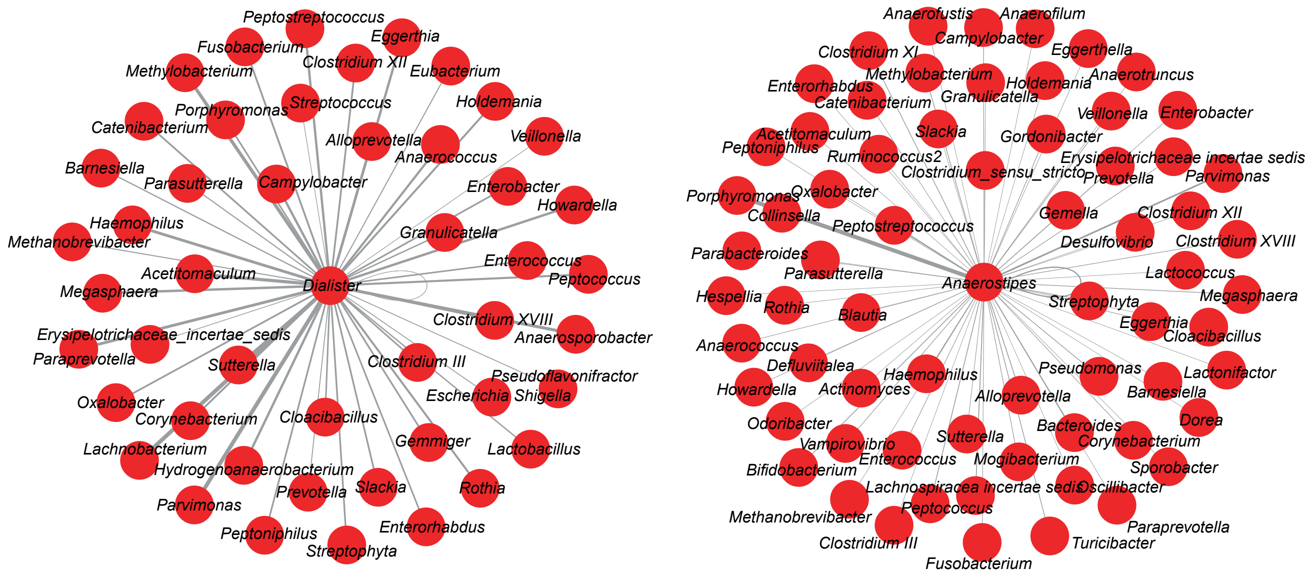

Supplement: Supplementary Figure S1 — Relationship alterations (RAs) involved by two microbes without abundance change. The RAs involved by Dialister (A) and Anaerostipes (B). [file mmc1.pdf]
